# Antibacterial activities of *Beilschmiedia obscura* and six other Cameroonian medicinal plants against multi-drug resistant Gram-negative phenotypes

**DOI:** 10.1186/1472-6882-14-241

**Published:** 2014-07-14

**Authors:** Aimé G Fankam, Jules R Kuiate, Victor Kuete

**Affiliations:** 1 Department of Biochemistry, Faculty of science, University of Dschang, P.O. Box 67, Dschang, Cameroon

**Keywords:** Antibacterial activity, *Beilschmiedia obscura*, Gram-negative bacteria, Multi-drug resistance, Efflux pumps, Medicinal plants

## Abstract

**Background:**

The rapid spread of bacteria expressing multi-drug resistance propels the search for new antibacterial agents. The present study was designed to evaluate the antibacterial activities of the methanol extracts from *Beilschmiedia obscura* and six other Cameroonian plants against a panel of twenty nine Gram-negative bacteria including Multi-drug resistant (MDR) phenotypes.

**Methods:**

The phytochemical investigations of the extracts were carried out according to the standard methods and the liquid micro-dilution assay was used for all antibacterial assays.

**Results:**

Phytochemical analysis showed the presence of alkaloids in all studied extracts. Other chemical classes of secondary metabolites such as anthocyanines, anthraquinones flavonoids, saponins, tannins, sterols and triterpenes were selectively detected in the extracts. The extract from the fruits of *Beilschmiedia obscura*, *Pachypodanthium staudtii* leaves and *Peperomia fernandopoiana* (whole plant) displayed the best spectrum of activity with MIC values ranging from 16 to 1024 μg/mL against at least 65% and above of the tested bacteria. The extract from *Beilschmiedia obscura* was the most active with MIC values below 100 μg/mL against ten of the tested bacteria. This extract also showed MBC values below 1024 μg/mL against 55.17% of the studied microorganisms. Phenylalanine arginine β-naphthylamide (PAβN) significantly modulated the activities of extracts from the leaves and fruits of *Pachypodanthium staudtii* and *Beilschmiedia obscura* respectively, by increasing their inhibitory activity against *Klebsiella pneumoniae* KP55 strain at least four fold.

**Conclusion:**

The overall results of the present investigation provide information for the possible use of the methanol extracts of the studied plant species, especially *B. obscura* to fight infectious diseases caused by Gram-negative bacteria including MDR phenotypes.

## Background

Bacterial chemo-resistance is a worrisome health concern worldwide today [1-3]. The development of multi-drug resistant (MDR) bacteria has severely compromised the efficacy of antimicrobial weapons and has dramatically increased the frequency of therapeutic failure [4]. Several reports highlighted the increased hospital dissemination of the bacterial strains expressing drug efflux mechanism [5,6]. Among the known efflux mechanisms of resistances in Gram-negative bacterial strains, Resistance-Nodulation-cell Division (RND) pump is one of the most occurring systems [7]. A number of chemicals such as phenylalanine arginine β-naphthylamide (PAβN), 1-(1-naphthylmethyl)-piperazine, some quinolone derivatives [8] as well as some natural products like reserpine [9] have been found to inhibit bacteria efflux pumps. The problem of bacterial resistance to commonly used antibiotics then shifted attention towards the discovery of new natural antibacterial compounds. Plants commonly used as herbal medicine may be a source of antibacterial, antifungal and antiviral activities [10-12]. In Cameroon, there is a rich tradition of using medicinal plants for the treatment of various infectious diseases, inflammations, injuries, and other diseases [13,14]. The aim of the investigation was to determine the antibacterial effects of twelve methanol crude extracts belonging to seven Cameroonian medicinal plants namely *Peperomia fernandopoiana* C.DC. (Piperaceae); *Cinchona succirubia* Par. Ex Klotzsk. (Rubiaceae), *Pachypodanthium staudtii* Engl & Diels (Annonaceae), *Vepris soyauxii* Engl. (Rutaceae), *Crassocephalum biafrae* (Oliv. & Hiern) S. Moore; *Beilschmiedia obscura (Staph). Engl.* (Lauraceae) and *Entada gigas* (Linn) Fawcelt & Rendle (Mimosaceae) against a panel of MDR Gram-negative bacteria expressing active efflux pumps. Most of these plant species or their related genus are known for their antimicrobial properties (Table 1). The role of efflux pumps was also investigated using pump-deleted bacteria and the efflux pump inhibitor (EPI) PAβN.

**Table 1:** Plants used in the present study and evidence of their bioactivities

**Samples, (family), and herbarium number**^**a**^	**Traditional treatment**	**Area of plant collection**	**Known bioactive (or potentially active) compounds**	**Screened activity for crude plant extracts and bioactive compounds**
*Vepris soyauxii* Engl. (Rutaceae) 18394 SFR/Cam	Fibromyomes [15], stomac ache and malaria [16,17].	Melon, Littoral region of Cameroon	Maculin, flindersiamin, skimmianin, (-) ribalinin, (+)-isoplatydesinin and araliopsin [17].	Stem bark extracts reduced sex hormones and some sperm parameters in male albinos rats [16].
*Peperomia fernandopoiana* C.DC. (Piperaceae); 7171 SRF/Cam	Gastrointestinal troubles and sterility (personal information)	Lebialem, South West region of Cameroon	**/**	
*Pachypodanthium staudtii* Engl & Diels (Annonaceae), 23170 SFR/Cam	Chest pain, tumors [18]; toothache [19]; bronchitis [20] and oedemas [21].	Ebolowa, Sud region of Cameroon	Pachypodol, 2,4,5-Trimethoxystyrene, Pachypophyllin, pachypostaudins A and B [21]; pachypodanthine [22]; Pachypophyllin and pachysontol [23]; staudin [24]; Sabinene, β -elemene, E- β -caryophyllene, β -selinene, β -bisabolene, δ -cadinene, 2,4,5-trimethoxy-1-vinylbenzene [25].	Hexane fraction protects legume seeds from bruchid insects: *Acanthoscelides obtectus* Say on common bean (*Phaseolus vulgaris*) and *Callosobruchus maculatus* on cowpea *(Vigna unguiculata)*[26].
				The flavonoid Pachypodol or 3,7,3’-tri-O-methylquercetin) showed antiviral activity against the polio-virus [21,27].
*Crassocephalum biafrae* (Oliv. et Hiern) S.Moore 43562 HNC	Diabetes, pulmonary defects ([28,29]; bleeding, sore eyes, cough, heart troubles, rheumatic pain, oedemas [30] and women infertility [31,32].	Bandja, Haut-Nkam; West region of Cameroon	Biafraecoumarins A, B, and C [33].	Biafraecoumarins A, B, and C exhibit low to significant antimicrobial activities against *E. coli, B. subtilis, S. aureus, P. picketti, T. longifusus, A.flavus, M.canis, F.solani, C.albicans, and C.glabrata*[33].
				The evidence on the puberty onset induction and ovarian folliculogenesis effect of the aqueous extract in immature female rat have been demonstrated [34].
*Entada gigas* (Linn) Fawcelt et Rendle (Mimosaceae) 5861 SRF/Cam	Microbial infections (Personal information)	Foreke-Dschang, West region of Cameroon	**/**	**/**
*Cinchona succirubia* Par. Ex Klotzsk. (Rubiaceae) 25851 SRF/Cam	Malaria [35,36].	Foreke-Dschang, West region of Cameroon	Quinine [36]	Evident antiplasmodial activity against chloroquine resistant *Plasmodium falciparum* and a total chemosuppression of parasitaemia in mice infected with *Plasmodium berghei* have been demonstrated [35].
*Beilschmiedia obscura* (Staph).Engl.(Lauraceae)2102 SRFK	Gastrointestinal infection (personal information)	Dschang market, West region of Cameroon	Obscurine [37]	/

^a^(HNC), Cameroon National Herbarium; (SRF): *Société des réserves forestière du Cameroun*; ^b^(/): Not reported.

## Methods

### Plant materials and extraction

All medicinal plants used in the work were collected in different areas of Cameroon between January and April 2012. The plants were identified at the National Herbarium (Yaounde, Cameroon), where voucher specimens were deposited under the reference numbers (Table 1). The air-dried and powdered plant material was weighed (300 g) and soaked in 1 L of methanol (MeOH) for 48 h at room temperature. The filtrate obtained through Whatman filter paper No1was concentrated under reduced pressure in a vacuum to obtain the crude extracts. All crude extracts were kept at 4°C until further uses.

### Preliminary phytochemical investigations

The plant extracts were screened for the presence of major secondary metabolite classes such as alkaloids, anthocyanins, anthraquinones, flavonoids, phenols, saponins, sterols and triterpenes according to common phytochemical methods previously described by Harbone [38].

### Chemicals

Chloramphenicol (CHL) (Sigma-Aldrich, St Quentin Fallavier, France) was used as reference antibiotic (RA). *p*-Iodonitrotetrazolium chloride (INT) and phenylalanine arginine β-naphthylamide (PAβN) (Sigma-Aldrich) were used as bacteria growth indicator and efflux pumps inhibitor (EPI) respectively.

### Bacterial strains and culture media

MDR isolates (Laboratory collection) and reference strains (from the American Type Culture Collection: *Escherichia coli* ATCC8739 and ATCC10536; *Enterobacter aerogenes* ATCC13048; *Klebsiella pneumoniae* ATCC11296; and *Providencia stuartii* ATCC29916) of *Escherichia coli, Enterobacter aerogenes, Klebsiella pneumoniae, Enterobacter cloacae*, *Pseudomonas aeruginosa*, *and Providencia stuartii* were used. Their features were previously reported [39,40]. They were maintained at 4°C and sub-cultured on a fresh appropriate Mueller Hinton Agar (MHA) for 24 h before any antibacterial test.

### Microdilution assay for MIC and MBC determinations

The microdilution inhibitory concentration (MIC) of the seven plant extracts and chloramphenicol were determined using a rapid microdilution assay [41,42]. Briefly, the samples were first dissolved in 10% dimethyl sulfoxide (DMSO)/Mueller Hinton Broth (MHB). The solution obtained was then added to MHB and serially diluted two fold (in a 96-well microplate). One hundred microliters of inoculum (1.5 × 10^6^ CFU/mL) prepared in MHB were then added. The plates were covered with a sterile plate sealer and then agitated with a shaker to mix the contents of the wells and incubated at 37°C for 18 h. The final concentration of DMSO was less than 2.5%, and did not affect the microbial growth. Wells containing MHB, 100 μl of inoculum, and DMSO at a final concentration of 2.5% served as the negative control. The MIC of each sample was detected after 18 h of incubation at 37°C following addition of 40 μl INT (0.2 mg/mL) and incubation at 37°C for 30 min. The MIC was defined as the lowest sample concentration that prevented color change of the medium and that resulted in the complete inhibition of bacterial growth [43]. Viable bacteria reduced the yellow dye to a pink. The minimum bactericidal concentration (MBC) of the sample was determined by sub-culturing 50 μl of the suspensions from the wells which did not show any growth after incubation during MIC assays to 150 μl of fresh broth, and re-incubated at 37°C for 48 hours before re-evaluation. The MBC was defined as the lowest concentration of sample which completely inhibited the growth of bacteria [44,45]. Each assay was performed in three independent tests in triplicate.

The tested samples, the samples were also tested in the presence of PAβN at a final concentration of 20 μg/mL as previously described [46] against nine of the most resistant bacteria strains. The MIC was determined as described above.

## Results

### Phytochemical composition of the plant extracts

The results of the qualitative phytochemical analysis indicated that alkaloids were present in all plant extracts. Each of the studied plant extract contained at least two classes of secondary metabolites (Table 2).

**Table 2:** Extraction yields and phytochemical composition of the plant extracts

**Plant samples**	**Part used in this study and extraction yield (%)**	**Phytochemical composition**								
		**Alkaloids**	**Anthocyanines**	**Anthraquinones**	**Flavonoids**	**Phenols**	**Saponins**	**Tannins**	**Sterols**	**Triterpenes**
*Vepris soyauxii*	Leaves (6.17%),	+	+	-	+	+	+	+	+	+
	Stem bark (8.58%)	+	+	+	+	+	+	+	-	+
	Root bark (9.26%).	+	+	-	+	+	+	+	-	+
*Peperomia fernandopoiana*	Whole plant (7.28%)	+	+	-	+	+	-	+	+	-
*Crassocephalum biafrae*	Whole plant (4.86%)	+	-	-	-	-	-	-	+	-
*Entada gigas*	Leaves (5.86%)	+	-	-	-	+	-	+	-	-
*Cinchona succirubia*	Leaves (10.18%)	+	-	-	+	+	-	+	+	-
	Stem barks (10.96%)	+	+	+	+	+	-	+	-	-
*Beilschmiedia obscura*	Fruits (3.37%)	+	+	-	-	+	+	+	-	+
*Pachypodanthium staudtii*	Leaves (10%)	+	+	+	-	+	-	+	+	-
	Stems bark (9.4%)	+	+	+	+	+	-	+	+	+
	Root (6.25%).	+	+	+	+	+	+	+	-	+

(+): present; (-): absent.

### Antibacterial activity of the plant extracts

The data depicted in Table 3 show that all extracts were active on at least two bacterial strains with MIC values varying from 16 to 1024 μg/mL. Extracts from *P. staudtii* leave*s* and *P. fernandopoina* (whole plant) displayed the highest spectrum of activity, their inhibitory effects being observed against 72.41% (21/29) of the bacterial strains, followed by those from the fruits of *B. obscura* (65.52%), stem barks of *V. soyauxii, P. staudtii* (55.17%), *P. staudtii* stem bark and *V. soyauxii* leaves (51.72%). The extract from *B. obscura* showed the best activity with MIC values below 100 μg/mL recorded against ten of the tested bacteria. The MIC values of this extract were lower than those of choramphenicol against *K. pneumoniae* Kp55 and *E. aerogenes* EA27 strains. Other extracts exhibited weak activities against a limited number of strains studied. The Bactericidal activity of the extracts was mostly noted with the extract from *B. obscura*.

**Table 3:** Minimal inhibitory concentration (MIC) and minimal bactericidal (MBC) of the plant extracts and chloramphenicol on the studied bacterial species

**Bacterial strains**	**Tested samples, MIC and MBC in parenthesis (μg/mL)**												
	***P. fernandopoina *** **(Whole plant)**	***C. succirubia***		***P. staudtii***			***V. soyauxii***			***C. biafrae *** **(Whole plant)**	***B. obscura *** **(Fruits)**	***E. giga *** **(Leaves)**	**CHL**
		**Leaves**	**Stem bark**	**Leaves**	**Stem bark**	**Root**	**Leaves**	**Stem bark**	**Root bark**				
***E. coli***													
ATCC8739	256(-)	512(-)	-	1024(1024)	1024(-)	1024(1024)	-	256(512)	512(512)	-	16(128)	-	2(32)
ATCC10536	512(-)	1024(-)	-	512(-)	-	1024(-)	1024(-)	256(1024)	512(1024)	1024(-)	32(256)	1024(-)	<2(32)
AG100	-	1024(-)	1024(-)	512(-)	1024(-)	512 (512)	1024(-)	1024(-)		1024(-)	64(512)	-	16(128)
AG100A	512(-)	512(-)	-	512(-)	1024(-)	512(-)	1024(-)	-	-	512(-)	128(512)	1024(-)	<2(64)
AG100A_Tet_	512(-)	-	-	1024(-)	-	1024(-)	1024(-)	512(512)	1024(-)	-	1024(1024)	-	32(256)
AG102	1024(-)	-	1024(-)	1024(-)	1024(-)	-	1024(-)	-	-	-	1024(-)	1024(-)	32(>256)
MC4100	1024(-)	512(-)	-	128(1024)	1024(-)	-	512(512)	512(1024)	256(1024)	512(-)	128(1024)	256(1024)	32(>256)
W3110	512 (1024)	1024(-)	-	-	1024(-)	-	1024(-)	256(1024)	1024(-)	1024(-)	32(256)	512(-)	8(128)
***E. aerogenes***													
ATCC13048	-(-)	1024(-)	-	512 (1024)	1024(-)	-	-	1024(-)	-	-	32(256)	1024(-)	8(64)
CM64	1024(-)	-	-	-	-	-	-	-	-	-	-	-	>256
EA27	1024(-)	-	-	1024(-)	1024(-)	512(-)	1024(-)	1024(-)	1024(-)	-	32(512)	-	64(256)
EA3	1024(-)	1024(-)	-	1024(-)	-	512(-)	-	-	512(-)	1024(-)	-	1024(-)	128(>256)
EA289	-	-	-	1024(-)	-	-	-	-	-	-	-	-	128(>256)
EA298	1024(-)	-	-	1024(-)	-	-	-	-	-	-	-	-	128(>256)
EA294	512(-)	1024(-)	-	256(512)	1024(1024)	1024(1024)	512(1024)	512(-)	-	-	64(-)	-	16(64)
***E. cloacae***													
ECCI69	1024(-)	-	-	-	-	-	-	-	-	-	-	-	>256
BM47	-	-	-	-	-	-	-	-	-	-	-	-	>256
BM67	-	-	-	-	-	-	-	-	-	-	-	1024(-)	>256
***K. pneumoniae***													
ATCC11296	256(1024)	1024(-)	-	256(1024)	512(-)	1024(1024)	1024(-)	256 (1024)	512 (1024)	-	64(256)	1024(-)	4(64)
KP55	512(512)	-	-	512 (1024)	1024(-)	512(-)	1024(1024)	1024(1024)	512(1024)	1024(-)	32(128)	-	64(256)
KP63	512(-)	1024(-)	-	1024(-)	-	1024(-)	1024(-)	1024(-)	-	1024(-)	512(1024)	-	64(256)
K24	1024(-)	-	-	-	-	-	-	-	-	-	-	-	32(256)
K2	512(-)	-	-	512(512)	512 (1024)	512(-)	1024(-)	-	-	512(-)	128(1024)	-	16(256)
***P. stuartuii***													
**ATCC29916**	1024(-)	1024(-)	-	1024(-)	1024(-)	512(-)	1024(-)	512(-)	512(-)	512(-)	64(256)	512(512)	16(128)
NEA16	-	-	-	-	-	-	-	-	-	-	-	-	64(256)
**PS2636**	1024	1024(-)	-	256(1024)	512(-)	512(-)	1024(-)	128(1024)	512(-)	1024(-)	128(512)	-	32(256)
**PS299645**	-	1024(-)	-	1024(-)	1024(-)	512(-)	-	512(-)	512(-)	1024(-)	128(512)	-	32(256)
***P. aeruginosa***													
PA01	1024(-)	-	-	1024(-)	-	1024(1024)	-	1024(-)	-	-	1024(-)	512(-)	32(256)
PA124	-	-	-	-	-	-	-	-	-	-	-	-	64(>256)

-MIC or MBC not detected at 1024 μg/mL. **()**: MBC of the tested samples (μg/mL). CHL: chloramphénicol.

### Role of efflux pumps in susceptibility of Gram-negative bacteria to the tested plant extracts

When combined, PAβN modulated significantly the activities (by decreasing the MIC values at least four times) of the extract from *P. staudtii* leaves and *B. obscura* on *K. pneumoniae* Kp55 strain. Therefore, PAβN in general had little or no effects on the increase of the activities of the tested plant extracts. It improved the activity of chloramphenicol on MDR bacteria used (Table 4).

**Table 4:** MIC of tested plant extracts in the absence and presence of PAβN against the studied bacterial strains

**Bacterial strains**	**Tested samples and MIC (μg/mL)**												
	***P. Fernandopoina *** **(Whole plant)**	***C. succirubia***		***P. staudtii***			***V. soyauxii***			***C. biafrae *** **(Whole plant)**	***B. obscura *** **(Fruits)**	***E. giga *** **(Leaves)**	**CHL**
		**Leaves**	**S. bark**	**Leaves**	**S. bark**	**Root**	**Leaves**	**S. bark**	**R. bark**				
***E. coli***													
AG100A_Tet_	1024 (512)	- (-)	- (-)	1024 (-)	- (-)	1024 (1024)	1024 (1024)	512 (1024)	1024 (1024)	- (-)	1024 (1024)	- (-)	*64 (≤2)*
AG102	1024 (1024)	- (-)	- (-)	1024 (1024)	- (1024)	- (-)	1024 (1024)	- (1024)	- (-)	- (-)	1024 (1024)	1024 (1024)	*32 (≤2)*
***E. aerogenes***													
CM64	- (-)	- (-)	- (1024)	- (-)	- (-)	- (-)	- (-)	- (-)	- (-)	- (-)	- (-)	- (-)	*>256 (16)*
EA289	- (-)	- (-)	- (-)	1024 (-)	- (-)	- (-)	- (-)	- (1024)	- (-)	- (-)	- (-)	- (1024)	*128 (16)*
***E. cloacae***													
ECCI69	1024 (1024)	- (1024)	*- (512)*	- (-)	- (-)	- (-)	- (-)	- (-)	- (1024)	- (-)	- (-)	- (-)	>256 (128)
***K. pneumonia***													
**KP55**	512 (512)	- (-)	- (-)	*512 (128)*	1024 (512)	512 (256)	1024 (1024)	1024 (1024)	512 (512)	1024 (-)	*32 (≤8)*	- (-)	*64 (4)*
K24	1024 (1024)	- (-)	- (1024)	- (-)	- (-)	- (-)	- (-)	- (-)	- (-)	- (-)	- (-)	- (-)	*32 (2)*
***P. stuartuii***													
NEA16	- (-)	- (-)	- (-)	- (1024)	- (-)	- (-)	- (-)	- (-)	- (-)	- (-)	- (-)	- (-)	*64 (4)*
***P. aeruginosa***													
PA124	- (-)	- (-)	- (-)	- (-)	- (-)	- (-)	- (-)	- (-)	- (-)	- (-)	- (-)	- (-)	*64 (8)*

(-): MIC not detected at up to 1024 μg/mL; (): MIC of samples in the presence of PAβN at 20 μg/mL. The MIC of PAβN was 128 μg/mL on *E. cloacae* ECCI69 and > 256 μg/mL on other bacteria.

CHL: chloramphenicol.

## Discussion

### Phytochemical composition of the plant extracts

The selective distribution of the secondary metabolites in the plant extracts may be due to the difference in the plant genus and family or the plant parts used. In fact, the presence of a particular metabolite can be influenced by the metabolisms which take place in the different plant parts. Compounds belonging to alkaloids as well as phenolics and terpenoids are well documented for their antibacterial activities [11]. Their presence in the studied extracts could therefore explain the observed activities.

### Antibacterial activity of the plant extracts

Phytochemicals are routinely classified as antimicrobials on the basis of susceptibility tests that produce MIC in the range of 100 to 1000 μg/mL [47]. Moreover, for crude extract, antimicrobial activity is considered to be significant if MIC values are below 100 μg/mL and moderate when 100 < MIC < 625 μg/mL [11]. Therefore, the activity recorded with *B. obscura* against ten of tested bacteria strains namely *E. coli* (ATCC10536, AG8739, AG100, W3110), *K. pneumoniae* ATCC11296, KP63), *E. aerogenes (*ATCC13048, EA27 and EA294) and *P. stuarti* ATCC29916 can be considered important. If we considered the alternative criteria described by Fabry *et al. *[48], where extracts having MIC values below 8000 μg/mL have noteworthy antimicrobial activity, the overall activity recorded with most of the studied extracts can be considered important, notably the extracts from *P. fernandopoina, B. obscura, V. soyauxii* and *P. staudtii* leave*s.* When analysing the MIC and MBC results for the crude extract, MBC/MIC ratios lower than 4 were noted with most of the studied samples, suggesting that their killing effects could be expected. Therefore, the extract from *B. obscura* displayed in many cases, a bacteriostatic effect (MBC/MIC > 4) [49]. To the best of our knowledge, the antibacterial activity of the plant extracts used is being reported herein for the first time, particularly towards MDR bacteria. Nevertheless, the antimicrobial potentials of the related genus for the most active plants have been demonstrated, particularly those of genus *Beilschmiedia*. Chouna *et al*. [50] demonstrated that compounds like beilschmiedic acid C from *B. anacardioides* were significantly active against *Bacillus subtilis*, *Micrococcus luteus* and *Streptococcus faecalis. Beilschmiedia cinnamomea* was previously demonstrated to have significant to moderate activities (64–1024 μg/mL) against the tested MDR bacteria [39]. Some compounds previously isolated from the genus *Beilschmiedia* and belonging to alkaloids, phenols, saponines, sterols and triterpenoids [50-52] were reported to possess antimicrobial activities [53]. The genus *Beilschmiedia* is also known traditionally to possess antimicrobial activities [53]. The fruits of *B. obscura* used herein are also used as soup ingredient in Cameroun [54]. This highlights its importance in the control of microbial infections and mostly those involving MDR phenotypes. Compounds belonging to alkaloids, flavonoids, sterols and triterpenoids classes previously isolated from *P. staudtii *[21-25] may be responsible for their observed activities. Bioactive alkaloids like araliopsin were previously isolated from *V. soyauxii *[17].

To assess the implication of efflux pumps in the susceptibility of Gram-negative bacteria to the tested plant extracts, PAβN a potent inhibitor of RND efflux systems and particularly active on AcrAB–TolC (of Enterobaceriaceae) and MexAB–OprM (of *Pseudomonas species*) [8,55] has been used at a concentration of 20 μg/mL. This concentration had no intrinsic effect on the bacteria as previously determined [46,56]. A significant increase of the antibacterial activities of the extract from *P. staudtii* and *B. obscura* was observed against resistant bacteria *K. pneumoniae* Kp55 strain, showing that one or more active compounds present in these plant extracts could be substrate(s) of efflux pumps of this bacteria. However, little or no increase of activities observed with the remaining extracts in the presence of EPI may be an indication that either secondary metabolites of these extracts are not active against the studied bacteria or that RND efflux pumps are not the main resistance mechanisms involved. The tested bacteria are good models in investigating MDR as they expressed active efflux pumps as observed when choramphenicol was tested in the presence of PAβN.

## Conclusions

The investigation provided informative data for the use of the crude medicinal plant extracts tested, especially those from *Beilschmiedia obscura, Peperomia fernandopoiana* and *Pachypodanthium staudtii* in the fight against MDR bacteria. The isolation of active constituents from these plants will further be performed in order to identify their active antibacterial ingredients.

## Competing interests

The authors declare that they have no competing interests.

## Authors’ contributions

AGF carried out the study; VK designed the experiments. AGF and VK wrote the manuscript; VK and JRK supervised the work; VK provided the bacterial strains; all authors read and approved the final manuscript.

## Pre-publication history

The pre-publication history for this paper can be accessed here:

http://www.biomedcentral.com/1472-6882/14/241/prepub
